# Genotypic Evolution of *Klebsiella pneumoniae* Sequence Type 512 during Ceftazidime/Avibactam, Meropenem/Vaborbactam, and Cefiderocol Treatment, Italy

**DOI:** 10.3201/eid2911.230921

**Published:** 2023-11

**Authors:** Gabriele Arcari, Federico Cecilia, Alessandra Oliva, Riccardo Polani, Giammarco Raponi, Federica Sacco, Alice De Francesco, Francesco Pugliese, Alessandra Carattoli

**Affiliations:** Sapienza University of Rome, Rome, Italy (G. Arcari, F. Cecilia, A. Oliva, R. Polani, G. Raponi, A. De Francesco, F. Pugliese, A. Carattoli);; Azienda Ospedaliero-Universitaria Policlinico Umberto I, Rome (A. Oliva, G. Raponi, F. Sacco, F. Pugliese)

**Keywords:** *Klebsiella pneumoniae*, ST512, sequence type 512, carbapenemase, KPC-3, KPC-31, KPC-154, inhibitor-resistant variants, VIM-1, ceftazidime, avibactam, meropenem, vaborbactam, cefiderocol, fosfomycin resistance, colistin resistance, *Acinetobacter baumannii*, *Providencia stuartii*, carbapenemase co-production, outer membrane porin disruption, siderophore disruption, CirA, antimicrobial resistance, bacteria, respiratory infections, Italy

## Abstract

In February 2022, a critically ill patient colonized with a carbapenem-resistant *K. pneumoniae* producing KPC-3 and VIM-1 carbapenemases was hospitalized for SARS-CoV-2 in the intensive care unit of Policlinico Umberto I hospital in Rome, Italy. During 95 days of hospitalization, ceftazidime/avibactam, meropenem/vaborbactam, and cefiderocol were administered consecutively to treat 3 respiratory tract infections sustained by different bacterial agents. Those therapies altered the resistome of *K. pneumoniae* sequence type 512 colonizing or infecting the patient during the hospitalization period. In vivo evolution of the *K. pneumoniae* sequence type 512 resistome occurred through plasmid loss, outer membrane porin alteration, and a nonsense mutation in the *cirA* siderophore receptor gene, resulting in high levels of cefiderocol resistance. Cross-selection can occur between *K. pneumoniae* and treatments prescribed for other infective agents. *K. pneumoniae* can stably colonize a patient, and antimicrobial-selective pressure can promote progressive *K. pneumoniae* resistome evolution, indicating a substantial public health threat.

The number of deaths caused by antimicrobial resistance was estimated at 1.27 million worldwide in 2019 (*1*), mainly attributed to 6 bacterial species: *Escherichia coli*, *Staphylococcus aureus*, *Klebsiella pneumoniae*, *Streptococcus pneumoniae*, *Acinetobacter baumannii*, and *Pseudomonas aeruginosa*. To reinforce the antimicrobial drug pipeline, the European Union’s European Medicines Agency authorized the clinical use of ceftazidime/avibactam (CZA) in 2016, meropenem/vaborbactam (MVB) in 2018, and cefiderocol (FDC) in 2020. Although those drugs all belong to the β-lactams class, substantial differences in mechanisms of action and antimicrobial spectra exist among them. CZA is a third-generation cephalosporin (ceftazidime) combined with a diazabicyclooctane β-lactamase inhibitor (avibactam). Avibactam prevents class A (including *K*. *pneumoniae* carbapenemase [KPC]), class C, and some class D β-lactamases from hydrolyzing ceftazidime, restoring ceftazidime activity in KPC- and oxacillinase-48–producing Enterobacterales but not in metallo-β-lactamase producers (*2*). MVB is a carbapenem (meropenem) combined with a boronate β-lactamase inhibitor (vaborbactam); vaborbactam inhibits class A but not class D or B carbapenemases (*3*). FDC is a siderophore cephalosporin that has a catechol moiety on the C-3 side chain (*4*), which can form a chelating complex with ferric iron; thus, FDC is subject to active transport through the iron transport system, including TonB-dependent receptors (*5*). In addition, FDC is highly stable against β-lactamase activity (*4*,*5*). 

In the past decade, Italy has seen a large increase in cases of carbapenem-resistant *K. pneumoniae* (*1*), mainly from 3 major KPC-producing (*6*) sequence types (STs): 101, 307, and 512 (*7*). In settings highly endemic for KPC-producing Enterobacterales, the selection of CZA-resistant, KPC-producing variants is of great concern (*8*,*9*). FDC resistance is not a 1-dimensional phenomenon (*10*); mutations in siderophore receptors (*11*), as well as in variants of β-lactamases KPC-2, CMY-2, CTX-M-15, and NDM-1, can confer FDC resistance (*12*). Nonetheless, *cirA* siderophore disruption substantially hinders bacterial fitness (*13*), and β-lactamase evolution contributing to FDC resistance typically comes at the price of functional trade-offs against other β-lactams (*12*). Under FDC treatment, in vivo resistance has been reported sporadically in *Enterobacter cloacae* (*14*) and *Escherichia coli* (*15*).

We describe a patient in Rome, Italy, who was colonized by carbapenem-resistant *K. pneumoniae* ST512. We integrated whole-genome sequencing, clinical, and microbiologic data to reconstruct the evolution of *K. pneumoniae* antimicrobial resistance in this patient after treatments for respiratory tract infections caused by different bacteria. 

## Methods

### Case Report

In February 2022, a 62-year-old patient who was positive for SARS-CoV-2 was transferred from a long-term care facility to Policlinico Umberto I (PUI) in Rome. The patient was hospitalized for 95 days initially in the COVID-19 intensive care unit (ICU) and then in the general ICU until death, which was caused by gastrointestinal bleeding. The patient’s medical history was notable for bipolar disorder, obesity, inflammatory bowel disease, type 2 diabetes, respiratory failure, chronic kidney failure, and heart failure. Six months before transfer to PUI, the patient had a percutaneous endoscopic gastrostomy performed because of severe gastrointestinal bleeding caused by underlying inflammatory bowel disease. During the patient’s hospitalization at PUI, we isolated a total of 5 different *K. pneumoniae* strains from patient rectal swab samples and respiratory tract specimens.

### Isolation of *K*. *pneumoniae* Strains and Susceptibility Testing

We identified the 5 *K. pneumoniae* strains by matrix-assisted laser desorption/ionization time-of-flight mass spectrometry (Bruker Daltonik GmbH, https://www.bruker.com). We identified carbapenemase genes by PCR using the GeneXpert system (Cepheid, https://www.cepheid.com) and used the lateral flow immunochromatography systems (NG-Test CARBA 5; Hardy Diagnostics, https://www.hardydiagnostics.com). We tested antimicrobial drug susceptibility by using the MicroScan WalkAway system (Beckman Coulter, Inc., https://beckman.com). We used gradient strips (Lioﬁlchem, https://www.liofilchem.com) to test MVB and CZA MICs and the Compact Antimicrobial Susceptibility Panel (ComASP; Liofilchem) to test FDC MICs.

### Whole-Genome Sequencing and Assembly

We performed whole-genome sequencing for each isolate. We purified genomic DNA by using the Isolate II Genomic DNA Kit (Bioline, https://www.bioline.com). We sent DNA to an external service to perform short-read Illumina sequencing (Illumina Inc., https://www.illumina.com). We obtained long reads by using the MinION Mk1C sequencing platform (Oxford Nanopore Technologies, https://nanoporetech.com). We extracted DNA for long reads by using the Wizard HMW DNA Extraction Kit (Promega, https://www.promega.com) and prepared libraries by using the Rapid Barcoding Kit 96; we sequenced libraries on R9.4.1 flow cells (Oxford Nanopore Technologies). We performed long-read assemblies by using Flye (*16*) with standard parameters. We integrated Illumina reads and Oxford Nanopore Technologies assemblies by using the Unicycler tool (*17*) in normal bridging mode and refined results by using the Bandage tool (*18*).

### Genomic and Phylogenetic Analyses

We analyzed single-nucleotide polymorphisms (SNPs) among the 5 sequenced genomes in this study by using the Snippy tool (https://github.com/tseemann/snippy). We annotated the 5 genomes by using Rapid Annotation using Subsystem Technology and compared by using SEED software (*19*).

We used Prokka software (*20*) to annotate 133 genomes belonging to ST512: 5 sequences from this study, 12 from a previous study performed at PUI (*8*), and 116 downloaded from the Pasteur Institute BIGSdb database (https://bigsdb.pasteur.fr/klebsiella). We analyzed the resulting general feature formats by using Roary software (*21*) to build a core genome alignment and generated a consensus phylogenetic tree by using 1,000 ultrafast bootstraps (*22*) in IQ-TREE 2 (*23*) and the transversion model plus base frequency plus proportion of invariable sites nucleotide substitution model (*24*). We visualized tree and metadata by using Microreact (*25*) and adjusted those data by using open source InkScape software (https://www.inkscape.org). We assessed all genomes for replicons by using PlasmidFinder (*26*), for capsular polysaccharide and lipopolysaccharide loci by using Kaptive (*27*), and for virulence and resistance gene content by using Kleborate (*28*) software.

### Cloning *bla*_KPC-154_ in pCR-Blunt II TOPO Vector

We cloned the novel β-lactamase *bla*_KPC-154_ allele from isolate 6099 into pCR-Blunt II TOPO-NeoR Vector and transformed TOP10 *E*. *coli* cells (both Thermo Fisher Scientific, https://www.thermofisher.com); we confirmed correctness of the cloned insert by Sanger sequencing. We tested the KPC-154 TOP10 *E. coli* clone for antimicrobial drug susceptibility by using the MicroScan system and measured CZA MICs by gradient tests (Lioﬁlchem) as previously described.

### OmpK36 Variant Modeling

We predicted structures of the outer membrane porin (Omp) K36 from isolate 0296 in silico by using Alphafold2 on the European Galaxy server (https://usegalaxy.eu) and analyzed those structures by using UCSF ChimeraX (*29*) for both the cartoon (ribbon) and surface images. We compared the structures with chain B in the crystal structure of OmpK36 from a *K. pneumoniae* ST258 clinical isolate that has a GD amino acid insertion (*30*). We used the Orientations of Proteins in Membranes database (*31*) to obtain spatial arrangements of the protein structures in lipid bilayers.

### Data Availability

We submitted the sequences of the strains analyzed in this study to GenBank. Whole-genome sequences are under Bioproject no. PRJNA992043 and complete plasmid sequences under accession nos. OQ096263 (pKpQIL-6099), OQ282880 (pIncA-6379), and OQ282881 (pIncA-0296).

### Ethics Approval

According to the hospital’s routine practice, the patient or his relatives gave informed consent to share data for research purposes during hospital admission. The study protocol was approved by the Ethics Committee of Azienda Ospedaliero-Universitaria Policlinico Umberto I (approval no. 109/2020).

## Results

### *K. pneumoniae* Strain Descriptions

**Figure 1:**
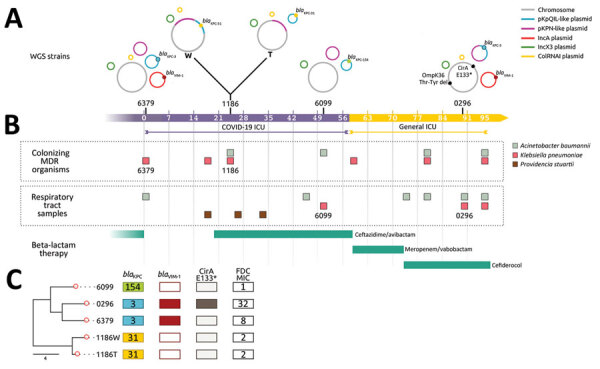
Timeline of colonization and infection of 1 patient by *Klebsiella pneumoniae* clones in study of genotypic evolution of *K.* *pneumoniae* sequence type 512 during ceftazidime/avibactam, meropenem/vaborbactam, and cefiderocol treatment, Italy. A) Schematic diagram of carbapenemase genes and plasmid content for *K. pneumoniae* strains 6379, 1186, 6099, and 0296; strain 1186 comprised 2 phenotypes: W and T colonies. Isolate collection day is indicated during 95 hospitalization days in either the COVID-19 (purple scale) or general (yellow scale) intensive care unit. Asterisk indicates the premature stop codon at position 133 (E133) in the catecholate iron outer membrane transporter CirA. B) Timeline of colonization and infection by *K. pneumoniae*, *Providencia stuartii*, and *Acinetobacter baumannii* as well as β-lactam therapies. C) Phylogenetic analysis used to compare the 5 *K. pneumoniae* strains isolated from the same patient. Core genome alignments were conducted for 5,215 core genes. KPC variants KPC-154, KPC-3, and KPC-31 are shown according to each strain; the *bla*_VIM-1_ gene was present in strains 0296 and 6379. Nonsense mutation in *cirA* was found in strain 0296, producing a premature stop codon (E133), indicated by an asterisk, in the protein. FDC MICs (mg/L) for each strain are shown. Scale bar indicates number of single-nucleotide polymorphisms in the core genome. FDC, cefiderocol; ICU, intensive care unit; KPC, *K.* *pneumoniae* carbapenemase; MDR, multidrug resistant; OmpK36, outer membrane porin K36; T, transparent; VIM, Verona integron-encoded metallo-β-lactamase; W, white; WGS, whole-genome sequencing.

On the first day of the patient's hospitalization at PUI, surveillance rectal swab samples tested positive for *K. pneumoniae* (strain 6379) that produced both KPC and Verona integron-encoded metallo-β-lactamase (VIM) (Figure 1). The patient had been treated with CZA at the long-term care facility for a previous carbapenem-resistant *K. pneumoniae* bloodstream infection; treatment was discontinued upon admission to PUI.

After 17 days of hospitalization, a respiratory tract infection caused by *Providencia stuartii* (>100,000 CFU/mL in bronchoalveolar lavage fluid) developed in the patient, accompanied by pleural effusion, which we treated with CZA. After 1 week of treatment, we isolated 2 CZA-resistant, meropenem-susceptible *K. pneumoniae* strains from patient rectal swab samples but not respiratory tract specimens; both strains produced KPC but not VIM and had dimorphic colony phenotypes: white (strain 1186W) and transparent (strain 1186T). Because of potential pleural infection, we maintained CZA therapy for ≈40 days, resulting in complete eradication of *P. stuartii* from the respiratory tract. 

On day 56, the patient was SARS-CoV-2 negative and was transferred to the general ICU at PUI. Respiratory tract samples were negative for *P. stuartii* but positive (>1,000 CFU/mL lavage fluid) for CZA-resistant *K. pneumoniae* (strain 6099) that produced KPC. CZA therapy was stopped, and MVB treatment was begun and continued until day 75. On day 75, respiratory tract samples tested negative for *K. pneumoniae* but positive for carbapenem-resistant *A. baumannii* (1,000 CFU/mL lavage fluid). Because of subsequent worsening respiratory conditions and chest imaging suggestive of new onset pneumonia, we replaced MVB therapy with FDC therapy (day 79) to treat *A. baumannii* pulmonary infection. On day 88, during FDC treatment, respiratory tract samples tested positive for both *A. baumannii* and *K. pneumoniae* (strain 0296) (1,000 CFU/mL lavage fluid each); strain 0296 was positive for KPC and VIM carbapenemases.

### Phylogeny, Antimicrobial Resistance, and General Features of ST512

**Figure 2:**
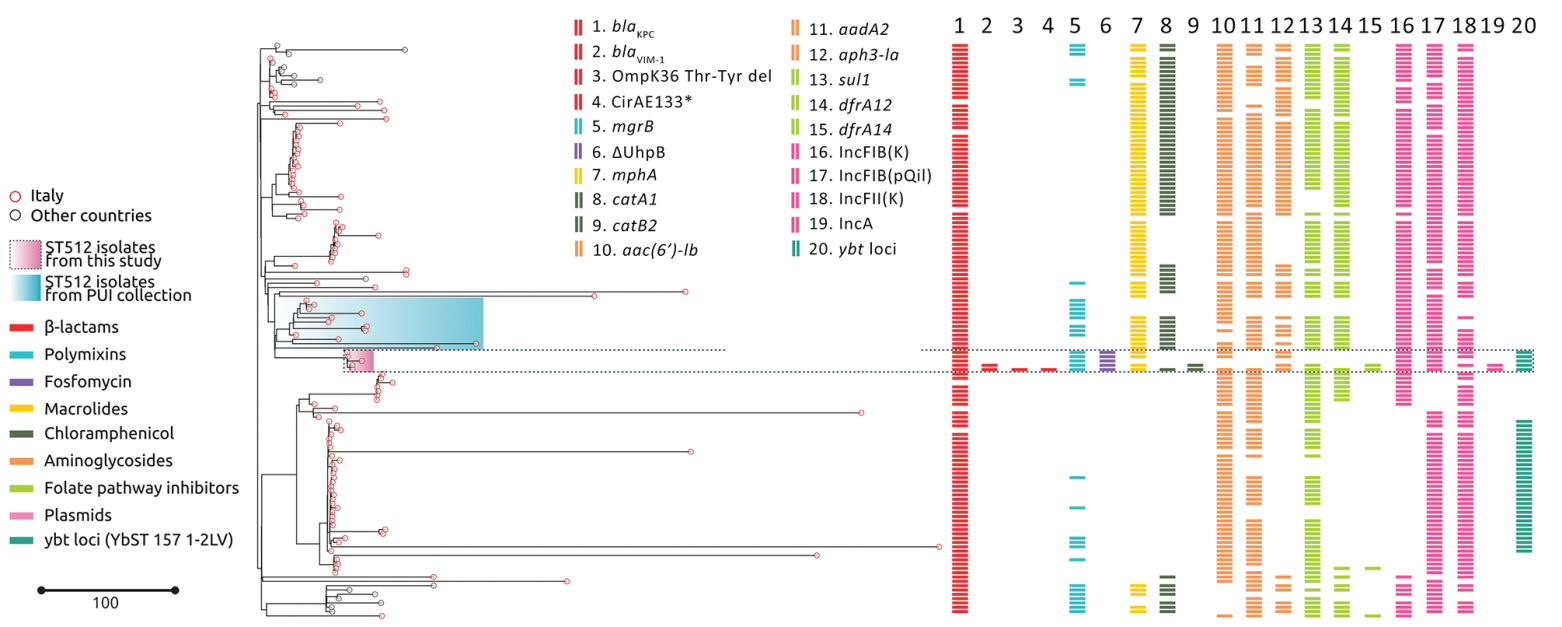
Phylogenetic analysis and genetic features of *Klebsiella pneumoniae* ST512 in study of the strain’s genotypic evolution during ceftazidime/avibactam, meropenem/vaborbactam, and cefiderocol treatment, Italy. Phylogenetic tree was constructed from 4,654 core gene alignments from a total of 133 *K. pneumoniae* ST512 genome sequences: 5 genomes sequenced in this study (pink shading), 12 genomes from the same hospital (pale blue shading), and 116 genomes downloaded from the Pasteur Institute BIGSdb database (https://bigsdb.pasteur.fr/klebsiella). Colors indicate resistance and acquired-resistance genes (or corresponding proteins) associated with carbapenemases, yersiniabactin, and chromosomal mutations within the different strains. The same color in the legend on the left indicates the expected resistance phenotype. Asterisk after CirA E133 indicates this mutation produced a premature stop codon. Scale bar indicates number of single-nucleotide polymorphisms in the core genome. Del, deletion; KPC, *K.* *pneumoniae* carbapenemase; PUI, Policlinico Umberto I; ST, sequence type; VIM, Verona integron-encoded metallo-β-lactamase.

We assigned the 5 *K*. *pneumoniae* isolates from the patient (6379, 1186W, and 1186T from rectal swab samples, 6099 and 0296 from respiratory tract samples) to ST512 by using in silico multilocus sequence typing of whole-genome sequences. We aligned the complete genomes of those strains against 116 ST512 genomes retrieved from the Pasteur Institute *K. pneumoniae* BIGSdb database (December 7, 2022) and 12 ST512 genomes previously identified at PUI (*8*,*32*). Phylogeny of 4,654 core genes showed how isolates from the patient clustered together in the same clade with ST512 strains isolated at PUI during 2018–2020 (Figure 2) and were separated by 0–11 SNPs (Appendix 1 Table 1).

All ST512 isolates are defined by conserved features: the *wzi* allele 154 (assigned to the KL107 capsule), typical of the main clade II of clonal group 258 (*33*); O1/O2v2 O locus, serotype O2afg; 35Q mutation in the chromosome-encoded SHV-11 β-lactamase (*28*); premature stop at codon 89 in the OmpK35 protein, in most isolates coupled with a GD amino acid insertion in the OmpK36 eyelet (*34*); and chromosome mutations in the *gyrA* (S83I) and *parC* (S80I) genes potentially conferring quinolone resistance (*35*). The 5 isolates in this study had additional common features diverging from the average ST512 strain: a SNP in the *mgrB* gene, which stops translation at aa 29 of the MgrB regulator protein, conferring resistance to colistin (*36*); a SNP in the *uhpB* gene, which stops translation at aa 206 of the UhpB protein (*37*), conferring resistance to fosfomycin; and the presence of the locus encoding yersiniabactin siderophore, designated as YbST157 (*38*). The yersiniabactin siderophore is not common in ST512 isolates because this locus could only be found in 31/133 (23%) ST512 genomes from the BIGSdb collection.

The 5 *K. pneumoniae* strains carried multiple acquired resistance genes (Appendix 2 Table 1). Plasmid content of the 5 ST512 strains from the patient was not homogeneous: strains 6379 (first strain isolated) and 0296 (last strain isolated) both carried the pKpQIL plasmid containing the *bla*_KPC-3_ gene and IncA plasmid carrying the *bla*_VIM-1_ gene (Figures 1, 2). Strains 1186W, 1186T, and 6099 lacked the IncA-*bla*_VIM-1_ plasmid.

### KPC Variants Conferring Resistance to Ceftazidime/Avibactam

On day 24, *K. pneumoniae* strains 1186W and 1186T colonized the patient (isolated from rectal swab samples). Those strains were negative for VIM-1 (loss of plasmid pIncA-*bla*_VIM-1_) but reached high levels of CZA resistance through the production of KPC-31. The *bla*_KPC-31_ gene replaced *bla*_KPC-3_ on an otherwise indistinguishable pKpQIL plasmid; the plasmid was integrated in the chromosome only in strain 1186W. The KPC-31 variant is characterized by a D179Y substitution in the Ω loop, previously shown to confer CZA resistance but restores carbapenem susceptibility (*39*).

On day 51, CZA-resistant *K. pneumoniae* strain 6099 caused a respiratory tract infection in the patient (Table). This strain hosted a pKpQIL plasmid encoding a novel KPC variant that was assigned by GenBank as KPC-154 (accession no. OQ096263) and was negative for the VIM-encoding IncA plasmid. Compared with KPC-3, KPC-154 has an RAPNKDDKYS amino acid duplication in position 263–273, corresponding to the 270 loop (*40*), but no differences are present in the Ω loop. When compared with an isogenic KPC-31–carrying strain, the strain carrying KPC-154 had higher MICs for several β-lactams (Appendix 2 Table 2).

**Table:** MICs of antimicrobial drugs for *Klebsiella pneumoniae* sequence type 512 strains analyzed in study of genotypic evolution of such strains during ceftazidime/avibactam, meropenem/vaborbactam, and cefiderocol treatment, Italy*

Strain	AZT	CZA†	FDC‡	MEM	MVB†	IMI	COL	FOS	AMK	GTM	CIP	SXT	TGC
6379	**>4**	**>256**	**8**	**32**	1.5	**>8**	**>4**	**>64**	**16**	≤2	**>1**	**>4/76**	2
1186W	**>4**	**32**	2	2	0.25	≤1	**>4**	**>64**	**>16**	≤2	**>1**	≤2/38	≤1
1186T	**>4**	**32**	2	4	0.25	≤1	**>4**	**>64**	**>16**	≤2	**>1**	≤2/38	≤1
6099	**>4**	**16**	1	**16**	0.5	**>8**	**>4**	**>64**	≤8	≤2	**>1**	≤2/38	≤1
0296	**>4**	**>256**	**32**	**32**	0.047	**>8**	**>4**	**>64**	≤8	≤2	**>1**	**>4/76**	≤1
EUCAST breakpoint	4	8	2	8	8	4	2	32	8	2	0.5	4	ND

*MICs are mg/L. Bold indicates resistance to antimicrobial drugs according to EUCAST breakpoints. AMK, amikacin; AZT, aztreonam; CIP, ciprofloxacin; COL, colistin; CZA, ceftazidime/avibactam; EUCAST, European Committee on Antimicrobial Susceptibility Testing (https://www.eucast.org); FDC, cefiderocol; FOS, fosfomycin; GTM, gentamicin; IMI, imipenem; MEM, meropenem; MVB, meropenem/vaborbactam; ND, not done; SXT, trimethoprim/sulfamethoxazole; TGC, tigecycline. †Tested by using the gradient strip method (Lioﬁlchem, https://www.liofilchem.com). ‡Tested by using the Compact Antimicrobial Susceptibility Panel broth microdilution method (Liofilchem).

### Cefiderocol Resistance in ST512 Isolates Co-Producing KPC and VIM 

**Figure 3:**
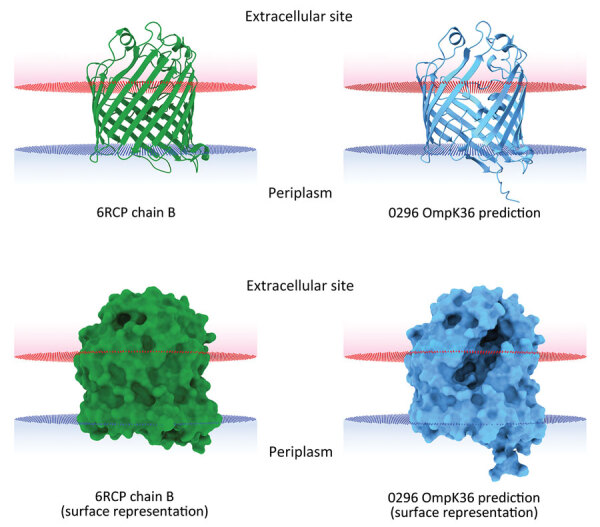
In silico 3-dimensional structure predictions for mutated OmpK36 porin in *Klebsiella* *pneumoniae* sequence type 512 strain 0296 in study of *K*. *pneumoniae* genotypic evolution during ceftazidime/avibactam, meropenem/vaborbactam, and cefiderocol treatment, Italy. The outer membrane porin OmpK36 from strain 0296 (blue) containing a 26 aa deletion from residue Thr263 through residue Tyr289 was modeled and compared with the model of reference OmpK36 chain B crystal structure from the Protein Data Bank (no. 6RCP; https://www.rcsb.org) (*30*). Both ribbon cartoon (top) and surface (bottom) models are shown. Structures for strain 0296 were obtained by using Alphafold2 on the European Galaxy server (https://usegalaxy.eu). Spatial arrangements of the porins in lipid bilayers were visualized by using the positioning of proteins in membranes web server in the Orientations of Proteins in Membranes database (*31*).

*K. pneumoniae* strain 0296 (pulmonary infectious agent, isolated on day 88) was genotypically related to strain 6379 (colonizer, isolated on day 1). Both strains had similar plasmid content, including pKpQIL carrying *bla*_KPC-3_ and pIncA carrying *bla*_VIM-1_ (Figures 1, 2). However, isolate 0296 carried a pKpQIL-pKPN fused plasmid carrying *catA1*, *dfrA12*, *aadA2*, *aph(3′)-Ia* genes, 2 copies of *mph*(A), *sul1*, and Δ*qacE* genes (Appendix 2 Table 1), and the previously described putative gene involved in iron acquisition (*fec*) (*41*), all of which were not present in plasmid pKPN from isolate 6379. Within the core genome, strain 0296 had 7 SNPs and 3 deletions when compared with strain 6379 (Appendix 1 Table 2); 1 SNP created a premature stop codon at position 133 in the iron transporter protein CirA (Table; Figure 1) (*42*). Strain 0296 showed 4-fold higher FDC MIC than strain 6379 (6379 FDC MIC = 8 mg/L; 0296 FDC MIC = 32 mg/L). Moreover, in strain 0296, a conservative in-frame deletion resulted in a novel mutation within OmpK36; the deletion was 26 aa (residues Thr263 to Tyr289) according to the reference OmpK36 crystal structure in the Protein Data Bank (no. 6RCP; https://www.rcsb.org) (*30*). The in silico predicted 3-dimensional structure of the mutated OmpK36 porin showed a deep lateral cave, probably favoring MVB permeability from the extracellular site into the periplasmic space (Figure 3). Strain 0296 carrying the mutated OmpK36 protein showed a 5-fold reduction in MVB MICs compared with strain 6379 (6379 MVB MIC = 1.5 mg/L; 0296 MVB MIC = 0.047 mg/L).

## Discussion

We report the in vivo evolution of antimicrobial resistance in *K. pneumoniae* high-risk ST512 strains (*43*) that colonized or infected a critical patient during a 95-day hospitalization. From both phenotypic and genotypic perspectives, each *K. pneumoniae* isolate had univocal characteristics. The rare mutation in the UhpABC signaling pathway conferred fosfomycin resistance by altering expression of the hexose phosphate transporter (*44*,*45*). Mutations in the *mgrB* gene, identified in the 5 strains isolated in this study, 8 strains isolated during 2018–2020 at the PUI (*8*), and globally in 37/133 (27.8%) whole-genome sequences from the Pasteur Institute ST512 collection, conferred colistin resistance. Moreover, the 5 strains from this study carried the yersiniabactin locus, a virulence factor rarely reported in ST512 clones. The yersiniabactin locus was identified in a *K. pneumoniae* ST512 cluster sampled in the framework of the SPARK project from a single city located in northern Italy during 2017–2018 (*46*). All of those factors support the hypothesis that a single *K. pneumoniae* clone was obtained from the patient first from rectal swab samples and subsequently from the respiratory tract, rather than antimicrobial drug treatment selecting 5 distinct strains.

The development of β-lactam resistance in *K*. *pneumoniae* ST512 strains is a critical concern. At the time of transfer to PUI from a long-term care facility, the patient was colonized by a carbapenem-resistant *K. pneumoniae* strain that produced KPC-3 and VIM-1. Although KPC-3 production by *K. pneumoniae* is a persistent phenomenon in Italy (*6*), the spread of VIM-producing *K. pneumoniae* is less common. However, we previously described an Enterobacterales infection outbreak at PUI caused by IncA plasmids encoding VIM (*47*). Therefore, we hypothesize that an elusive and untraced spread of VIM-producing Enterobacterales might exist in hospitals and long-term care facilities in Rome.

During the first 3 weeks of hospitalization, the patient was not treated with β-lactams. We introduced CZA therapy to treat a respiratory tract infection sustained by *P. stuartii*, a pathogen more frequently associated with urinary tract infections (*48*) but also related to respiratory tract infections (*49*,*50*). After 3 days of CZA treatment, a KPC-31–producing, CZA-resistant *K. pneumoniae* strain colonized in the patient and then evolved to an infection by a KPC-154–producing CZA-resistant variant. The KPC-154 clone had a wild type Ω loop but a 13 aa insertion in the 270 loop of the enzyme, a feature previously described in other KPC variants (*8*).

We used MVB to treat the KPC-154–producing *K. pneumoniae* infection, which eradicated the *K. pneumoniae* strain from respiratory tract samples. However, a respiratory tract infection sustained by *A. baumannii* developed in the patient, who we then treated with FDC. Under FDC treatment, the respiratory tract sample tested positive for *K. pneumoniae* ST512 that was identical to the first colonizing strain, producing both KPC-3 and VIM-1 carbapenemases. This result suggests that a mixed population of KPC/VIM–positive and KPC-positive/VIM-negative *K. pneumoniae* was present in the patient during the entire hospitalization period. VIM-1–negative strains prevailed under CZA treatment, whereas MVB and FDC treatments selected the expansion of VIM-1–producing strains, which likely remained in a hidden reservoir within the patient.

The reemerged KPC/VIM producer had a high MIC for FDC because of the loss-of-function mutation in the CirA siderophore receptor. CirA mutations have previously been associated with FDC resistance in an in vitro model of New Delhi metallo-β-lactamase–producing *K. pneumoniae* (*11*) and in vivo for *E. cloacae* (*14*) and *E. coli* (*15*). However, the KPC/VIM–producing *K. pneumoniae* strain showed increased susceptibility to MVB, probably associated with a novel OmpK36 structure showing a large protein deletion; the 3-dimensional porin structure predicted a large lateral cave that might increase permeability of MVB. Nonetheless, the mutant OmpK36 could also be interpreted as a compensatory mutation that promotes survival of the CirA-defective strain.

We used last resort β-lactam–based antimicrobial drugs (CZA, MVB, and FDC) to treat the patient but only used MVB to treat the respiratory tract infection sustained by the CZA-resistant ST512 *K. pneumoniae*; other antimicrobial drugs were administered to treat infections caused by *P. stuartii* or *A. baumannii*. This case report serves as a warning that cross-selection can occur between *K. pneumoniae* and treatments prescribed against other infective agents and that *K. pneumoniae* can stably colonize a patient for a long period, promoting progressive evolution of the resistome under increasing selective pressure.

Appendix 1 Data used in investigation of genotypic evolution of *Klebsiella pneumoniae* sequence type 512 during ceftazidime/avibactam, meropenem/vaborbactam, and cefiderocol treatment, Italy.

Appendix 2 Additional information for genotypic evolution of *Klebsiella pneumoniae* sequence type 512 during ceftazidime/avibactam, meropenem/vaborbactam, and cefiderocol treatment, Italy.
