# Supplementary material for: Genotypic Evolution of Klebsiella pneumoniae Sequence Type 512 during Ceftazidime/Avibactam, Meropenem/Vaborbactam, and Cefiderocol Treatment, Italy
Source: Emerg Infect Dis. 2023 Nov;29(11):2266–74. doi: 10.3201/eid2911.230921 (PMC10617348; doi:10.3201/eid2911.230921)
Supplement: Appendix 2 — Additional information for genotypic evolution of Klebsiella pneumoniae sequence type 512 during ceftazidime/avibactam, meropenem/vaborbactam, and cefiderocol treatment, Italy. [file 23-0921-Techapp-s2.pdf]

Article DOI: https://doi.org/10.3201/eid2911.230921

EID cannot ensure accessibility for supplementary materials supplied by authors. Readers who have difficulty accessing supplementary content should contact the authors for assistance.

Evolution of *Klebsiella pneumoniae* Sequence Type 512 during Ceftazidime/Avibactam, Meropenem/Vaborbactam, and Cefiderocol Treatment, Italy

Appendix 2

Appendix 2 Table 1. Sample type, day of isolate collection, resistance genes, and replicon content identified in *Klebsiella pneumoniae* sequence type 512 genomes analyzed in this study*

Isolate	Sample source	Collection day	β-lactamases	Other acquired resistance genes	Replicon content
6379	RS	1	bla_{KPC-3}, bla_{SHV-12}, bla_{TEM-1A}, bla_{VIM-1}	*aac(6')-Ib, aac(6')-Ib3, aadA1, aph(3')-XV, catB2, dfrA14, mph*(A), *ΔqacE, sul1*	ColRNAI, IncA, IncFIB(K), IncFII(K), IncFIB(pQil), IncX3
1186B	RS	24	bla_{KPC-31}, bla_{TEM-1A}	*aac(6')-Ib, aph(3')-Ia, mph*(A)	ColRNAI, IncFIB(K),† IncFII(K),† IncFIB(pQil),† IncX3
1186T	RS	24	bla_{KPC-31}, bla_{TEM-1A}	*aac(6')-Ib, aph(3')-Ia, mph*(A)	ColRNAI, IncFIB(K),† IncFIB(pQil), IncFII(K), IncX3
6099	RT	51	$bla_{KPC-154}$, bla_{TEM-1A}	None	IncFIB(K), IncFIB(pQil), IncX3
0296	RT	88	bla_{KPC-3}, bla_{SHV-12}, bla_{TEM-1A}, bla_{VIM-1}	*aac(6')-Ib, aac(6')-Ib3, aadA1, aadA2, aph(3')-Ia, aph(3')-XV, catB2, catA1, dfrA14, dfrA12, mph*(A),*mph*(A), *ΔqacE, ΔqacE, sul1, sul1*	ColRNAI, IncA, IncFIB(K),‡ IncFII(K),‡ IncFIB(pQil),‡ IncX3

*Virulence and resistance genes were identified by using Kleborate (*28*, main text) software and replicons were identified by using PlasmidFinder (*26*, main text). RS, rectal swab sample, RT, respiratory tract sample.
†Replicon integrated in the chromosome.
‡pKpQIL-pKPN fused plasmid.

Appendix 2 Table 2. MICs of antimicrobial drugs used to treat *Escherichia coli* recombinant clones carrying bla_{KPC} gene variants KPC-31 and KPC-154 from *Klebsiella pneumoniae* sequence type 512*

	MIC (mg/L)	
Antimicrobial drugs	KPC-31	KPC-154
AUG	≤8	>32
AZT	≤1	>4
CFZ	≤16	>16
CPE	≤0,5	2
CZA	>8	8
ETP	≤0.12	≤0.12
MEM	≤0.12	≤0.12
P/T	≤8	16

*Gene variants from *K. pneumoniae* were cloned into pCR-Blunt II TOPO-NeoR Vector (Thermo Fisher Scientific, https://www.thermofisher.com). AUG, amoxicillin/clavulanic acid; AZT, aztreonam; CFZ, cefazolin; CPE, cefepime; CZA, ceftazidime/avibactam; ETP, ertapenem; MEM, meropenem; P/T, piperacillin/tazobactam.
